# Thickening of Dorsal Foot Nerves: A Frequent Sonographic Finding in Asymptomatic Volunteers, Potentially Leading to False Positive Results

**DOI:** 10.3390/diagnostics16020303

**Published:** 2026-01-17

**Authors:** Veronika Vetchy, Tobias Rossmann, Paata Pruidze, Wolfgang Grisold, Wolfgang J. Weninger, Stefan Meng

**Affiliations:** 1 Department of Biomedical Imaging and Image-Guided Therapy, Medical University of Vienna, 1090 Vienna, Austria; veronika.vetchy@meduniwien.ca.at; 2 Center for Anatomy and Cell Biology, Medical University of Vienna, 1090 Vienna, Austria; tobias.rossmann@kepleruniklinikum.at (T.R.); paata.pruidze@meduniwien.ac.at (P.P.); wolfgang.weninger@meduniwien.ac.at (W.J.W.); 3 Department of Neurosurgery, Neuromed Campus, Kepler University Hospital, 4040 Linz, Austria; 4 Neurology Consultancy Unit, Division of Anatomy, Medical University of Vienna, 1090 Vienna, Austria; wolfgang.grisold@wfneurology.org; 5 Department of Radiology, Hanusch Hospital, 1140 Vienna, Austria

**Keywords:** nerve ultrasound, nerve thickening, asymptomatic thickening, anterior tarsal tunnel syndrome, deep peroneal nerve

## Abstract

**Objectives**: Compression neuropathies such as Anterior Tarsal Tunnel Syndrome are usually associated with focal thickening at the compression site. This study aimed to determine the frequency and location of thickenings of dorsal foot nerves in asymptomatic, healthy volunteers. We hypothesized that focal nerve thickening of dorsal foot nerves is a frequent finding in asymptomatic individuals and occurs at anatomically plausible locations, potentially limiting the specificity of ultrasound in the diagnosis of anterior tarsal tunnel syndrome. **Materials and Methods**: In this prospective study, the nerves at the dorsal foot were examined with ultrasound in 60 volunteers without clinical signs of neuropathy. Cross-sectional area (CSA) changes along the nerve course were assessed, their anatomical location recorded, and demographic data collected. **Results**: Focal deep peroneal nerve (DPN) thickening was observed in 45% of participants, with a median CSA of 2.14 mm^2^ (range: 0.84–5.16) and median length of 3.98 mm (range: 1.46–9.95). The most frequent site was the first tarsometatarsal joint (41%). Thickening occurred across all age groups. Superficial peroneal nerve (SPN) thickening was found in 13.3% of participants, primarily affecting the intermediate branch, with a median CSA of 1.82 mm^2^ and length of 3.02 mm. No thickening was observed in the sural nerve (SN). A strong correlation was found between CSA and length of DPN thickening (r = 0.67, *p* < 0.001). **Conclusions**: Asymptomatic, focal thickening of dorsal foot nerves, particularly the DPN, is a frequent sonographic finding in healthy volunteers. These findings highlight the potential for false-positive ultrasound results and the necessity of correlating imaging findings with clinical examination when evaluating for anterior tarsal tunnel syndrome and similar neuropathies.

## 1. Introduction

**Figure 1:**
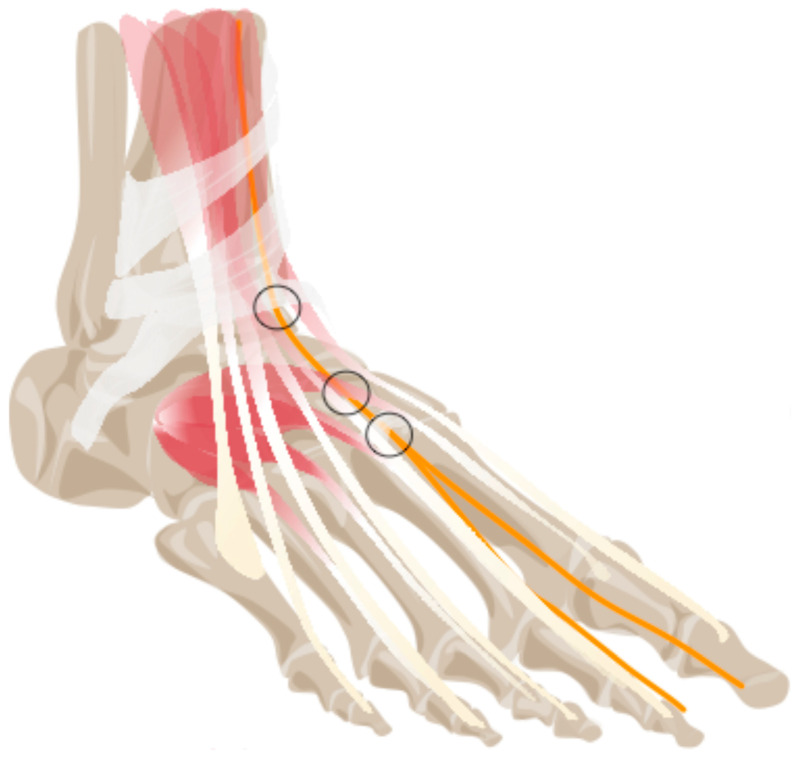
Anatomical schematic of the deep peroneal nerve at the dorsum of the foot. Circles indicate potential compression sites beneath the inferior extensor retinaculum, beneath the extensor hallucis brevis tendon, and at distal fascial constrictions near the first tarsometatarsal region.

Anterior tarsal tunnel syndrome (ATTS) is a compression neuropathy of the deep peroneal nerve (DPN) that occurs in the anterior tarsal tunnel (ATT) beneath the inferior extensor retinaculum, bounded by the extensor hallucis longus and extensor digitorum longus tendons [1,2]. Beneath the tendon of the extensor hallucis brevis, a continuation of the deep crural fascia binds the DPN and accompanying vessels between the first and second metatarsal bones and the first and second cuneiforms [3]. The DPN is said to be susceptible to compression at several key sites along its course through the ATT (Figure 1) [3,4]. Compression at these locations can result from various factors such as trauma, repetitive stress, anatomical variations, or space-occupying lesions [3]. Patients with ATTS typically present with pain and numbness in the first interdigital space or weakness and atrophy of the short extensor muscles of the foot, depending on the specific location of the compression site. Compression of the superficial peroneal nerve (SPN) is less often described. The compression site most mentioned is the lateral crural septum and the location of its transgression from the subfascial to the subcutaneous compartment, approximately 12 cm above the lateral malleolus [4,5].

The sural nerve (SN) is a primary sensory nerve that courses alongside the small saphenous vein and supplies the lateral rim of the foot as the lateral dorsal cutaneous nerve [6]. Non-traumatic injury of the SN is described similarly to the SPN at the level of its change from deep to superficial at the lateral border of the Achilles tendon. Studies have shown that the nerves of the dorsal foot, including the DPN, SPN, and SN can be examined using ultrasound [5,6,7,8,9].

Mechanical strain on a nerve due to compression or trauma leads to thickening of the internal and external epineurium and therefore an increase in the cross-sectional area (CSA) [10,11]. Those changes can be seen as thickening using ultrasound, which is usually interpreted as a pathologic finding [12,13].

In clinical practice focal thickening of the dorsal foot nerves, especially the DPN, can be frequently observed in asymptomatic individuals. However, their clinical relevance and potential implications for diagnosing or predicting an underlying pathology remain uncertain. Previous ultrasound studies have primarily focused on symptomatic patients with confirmed nerve entrapment [4,5,8,14], whereas systematic data on the prevalence and anatomical distribution of focal nerve thickening in asymptomatic individuals at the dorsum of the foot are lacking. This study set out to determine the frequency and location of thickening of dorsal foot nerves in asymptomatic volunteers in relation to adjacent anatomical landmarks.

## 2. Materials and Methods

### 2.1. Participants

For this prospective Study, a total of 60 healthy volunteers were prospectively recruited through a public announcement at our institution over the period of one year through convenient volunteer sampling. Included was any adult aged above the age of 18 years that gave written informed consent. All individuals with a history of comorbidities such as polyneuropathy, diabetes, previous surgery, or injuries of the foot as well as L5 radiculopathy were excluded. If only one foot was affected by surgery/injury, single foot participation was accepted. Given the exploratory nature of this study and the absence of prior data on the prevalence of dorsal foot nerve thickening in asymptomatic individuals, no formal a priori sample size calculation was performed.

### 2.2. Study Design

This study was performed in line with the principles of the Declaration of Helsinki. Approval was granted by the local ethics committee (ECS 1948/2022). All participants gave written informed consent to participate in the study. It must be noted that this dataset forms part of a larger research project. Further analyses addressing different research questions will be reported separately.

The authors declare that there is no conflict of interest or competing interests. No funding was received for this study.

Assessment of the sensory territory of the DPN, SPN, and SN was performed. Patients were labeled “asymptomatic” if clinical examination of the feet showed no abnormalities. Particular attention was given to the sensory palpable volume of the extensor digitorum brevis muscle to ensure intact motor function of the DPN.

All participants were examined for regular palpable arterial pulses, skin defects, and foot symmetry. Only asymptomatic participants were included.

### 2.3. Ultrasound Examination

**Figure 2:**
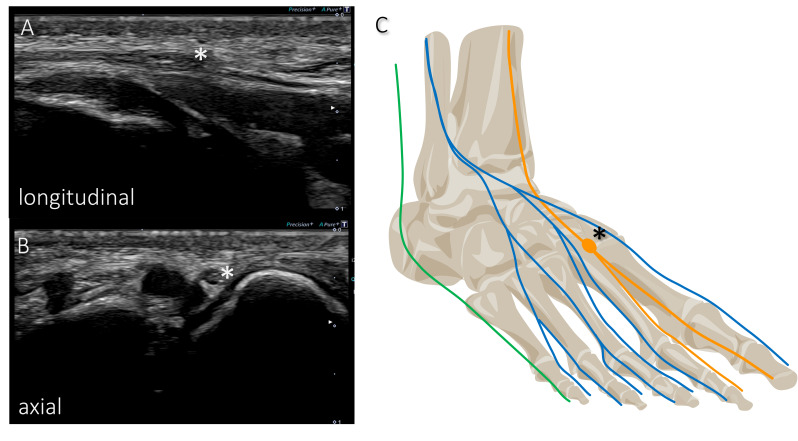
Ultrasound images of the thickened deep peroneal nerve in (**A**) longitudinal plane, (**B**) axial plane, and (**C**) a corresponding anatomical schematic diagram. Orange: deep peroneal nerve; blue: superfical peroneal nerve; green: sural nerve. The asterisk (*) depicts a thickened nerve segment at the level of the first tarsometatarsal joint.

All ultrasound examinations were performed by two radiologists experienced in peripheral nerve sonography using a standard clinical ultrasound system with a 22 MHz high-resolution transducer (Aplio i800 with i22LH8, Canon Medical Systems Europe B.V., Amstelveen, The Netherlands). The DPN was located at the distal lower leg and traced, following the medial terminal branch to the first webspace. The SPN was followed from the site of its fascial passage to the subcutaneous layer, also at the distal lower leg. Both the medial branch and the intermediate branch of the SPN were analyzed until the level of the head of the metatarsal bones. The SN was located behind the lateral malleolus and followed alongside the lateral foot margin. Nerve thickenings, nerve morphology, and the location in relation to the adjacent bony structures were noted. At nerve thickenings we measured the cross-sectional area (CSA) and length (Figure 2).

### 2.4. Statistical Analysis

Continuous variables were visually assessed using box plots and described as means and standard deviations if they were normally distributed, or otherwise, as medians and ranges. Categorical variables were described using frequency and percentage. Age groups were described using quartiles (Q1 ages 20–24 years, *n* = 15; Q2 ages 25–28, *n* = 15; Q3 ages 29–40, *n* = 15 and Q4 ages 41–78, *n* = 16). Thickenings were described for left and right feet separately or, when describing correlation coefficients, as averages and as maxima for thickenings in both feet. Bivariate correlations were described using Pearson correlation coefficients or Spearman correlation coefficients for skewed data distributions and their 95% confidence intervals. We used two-tailed analysis, and *p*-values were assumed significant when <0.05. Variables were shown stratified by sex and by age (using the median age).

## 3. Results

A total of 60 asymptomatic participants (33 female, 55%) with a median age of 28 years (range 20–78) were included. A total of 118 feet were examined sonographically, with bilateral assessments in 58 individuals and unilateral examination in two participants due to prior foot pathology of the excluded foot.

One was excluded due to a metatarsal bone fracture, and the other due to a history of recurrent gout. One potential participant was excluded because the affected side of a previous metatarsal bone fracture was unclear. Two participants (3.3%) had a history of arthritis, and one (1.7%) presented with a previously diagnosed flexible flatfoot (Table 1).

### 3.1. Deep Peroneal Nerve

Focal thickening of the DPN was observed in 30 participants (50.0%) at the dorsum of the foot. Of these, 21 participants (35.0%) had unilateral thickening, and 9 participants (15.0%) showed bilateral thickening. On a foot level, thickening was found in 20 of 59 left feet (33.9%) and 19 of 59 right feet (32.2%). The median cross-sectional area (CSA) of the thickened DPN segments was 2.03 mm^2^ (range: 0.84–5.16), and the median length was 3.53 mm (range: 1.46–9.95) (Table 2).

**Figure 3:**
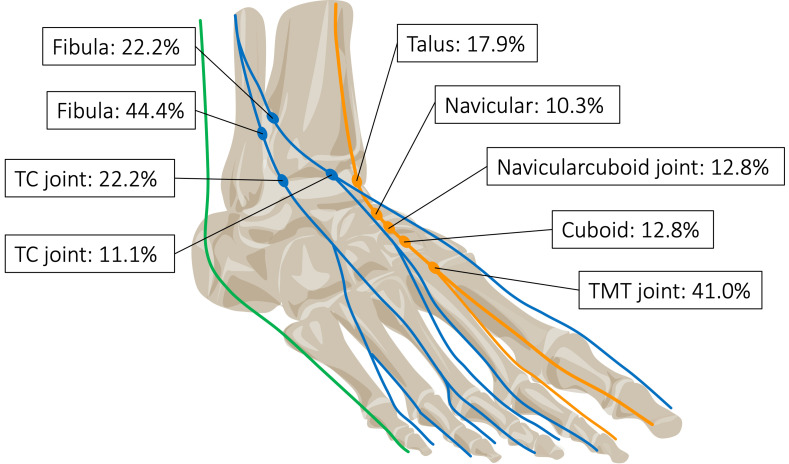
Frequency of nerve thickening by location: Thickening of the deep peroneal nerve (orange) was observed at the level of the talus, navicular bone, navicular-cuboid joint, cuboid bone, and the first tarsometatarsal (TMT) joint. The intermediate branch of the superficial peroneal nerve (blue) exhibited thickening at the crossing point with the fibula and at the level of the talocrural (TC) joint, while the medial branch showed thickening exclusively at the level of the TC joint. No thickening of the sural nerve (green) was found.

Focal DPN thickenings were most commonly localized at the first tarsometatarsal (TMT) joint, accounting for 16 of 39 thickenings (41.0%). Other common locations included the talus (*n* = 7, 17.9%), the cuboid bone (*n* = 5, 12.8%), the naviculocuboid joint (*n* = 5, 12.8%), and the navicular bone (*n* = 4, 10.3%) (Figure 3).

**Figure 4:**
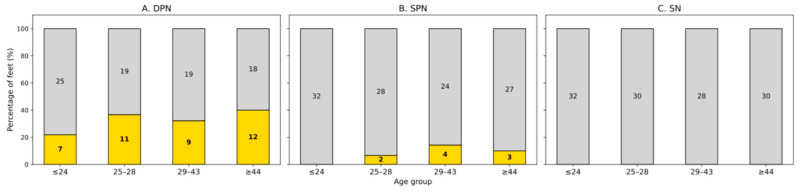
Occurrence of nerve thickening (per foot) (yellow) compared to no thickening (grey) of the deep peroneal nerve (**A**) and superficial peroneal nerve (**B**) sural nerve (**C**) with increasing age of participants grouped into quartiles.

To evaluate age-related patterns, participants were divided into quartiles. In Q1, 33.3% of participants showed unilateral and 6.7% bilateral thickening. In Q2, 33.3% of participants had unilateral and 20.0% had bilateral thickening. A total of 20.0% of participants in Q3 showed unilateral and 26.7% bilateral thickening. In Q4, 50.0% showed unilateral thickening, while 6.3% of participants showed bilateral involvement. These findings demonstrate a high prevalence of asymptomatic DPN thickening across all age groups, with a notable increase in unilateral findings among older participants. While bilateral thickening was most common in middle-aged individuals (Q3), the oldest participants (Q4) primarily showed unilateral thickening, suggesting an age-related shift in the laterality pattern of DPN changes (Figure 4).

**Figure 5:**
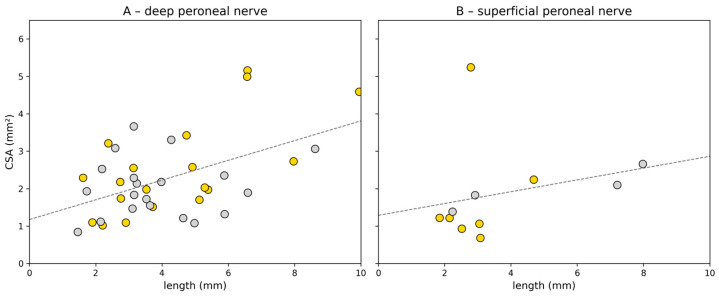
Correlation of cross-sectional area (CSA) and length of the thickenings the deep peroneal nerve (**A**) and superficial peroneal nerve (**B**). yellow = right foot; grey = left foot; dashed line indicates the linear regression.

The cross-sectional area (CSA) of the DPN thickenings showed a significant positive correlation with the length of the thickened segment (Pearson’s r = 0.67, 95% CI [0.35–0.86], *p* < 0.001), based on data from 27 feet. This indicates that thickenings with a larger CSA tended to extend over longer nerve segments (Figure 5).

### 3.2. Superficial Peroneal Nerve

Focal thickening of the SPN was detected in eight participants (13.3%). Of these, seven participants (11.7%) had unilateral thickening, and one participant (1.7%) exhibited bilateral thickening. On a foot level, thickening was found in 4 of 59 left feet (6.8%) and 5 of 59 right feet (8.5%). Thickening was more frequently observed in the intermediate branch of the SPN (six thickenings, 66.7%), compared to the medial branch (three thickenings, 33.3%). One participant exhibited bilateral thickening of the intermediate branch. No participant had focal thickening of both branches on the same foot, although in two cases, double thickenings were documented within the same branch on a single side (Table 2).

Within the intermediate branch, the most frequent site was the fibular crossing region, accounting for 4 cases (44.4%). The remaining 2 thickenings (22.2%) were located at the talocrural joint. In the medial branch, 2 thickenings (22.2%) were also found at the fibular crossing (11.1%), and 1 was observed at the talocrural joint (Figure 3).

The median CSA for the intermediate branch was 1.82 mm^2^ (range: 0.68–5.24), with a median length of 3.02 mm (range: 2.24–4.69). For the medial branch, the median CSA was 1.99 mm^2^ (range: 1.22–2.66), and the length was 5.68 mm (range: 1.85–7.98), based on a smaller number of measurements (*n* = 3). When combining all SPN thickenings from both branches (*n* = 9), a moderate but non-significant monotonic association was observed (Spearman’s ρ = 0.40, 95% CI [−0.31 to 0.85], *p* = 0.286).

In the youngest group (Q1), no participant showed thickening of the SPN (0/15, 0.0%). In Q2, unilateral thickening was found in 2 of 15 participants (13.3%). The third quartile (Q3) included One participant with bilateral and 2 with unilateral thickening (3/15 total, 20.0%; bilateral: 6.7%, unilateral: 13.3%). The oldest group (Q4) showed unilateral thickening in 3 of 16 participants (18.8%). No bilateral thickening was seen in this group. These findings suggest that when SPN thickening is present, it is predominantly unilateral. A trend toward increased frequency with age was observed, although the total number of cases remains low across all groups (Figure 4).

### 3.3. Sural Nerve

We did not find any thickenings of the investigated segment of the sural nerve at the lateral, dorsal foot in the participants of this study.

## 4. Discussion

In this study, focal thickening of the DPN was observed in 45% of participants, and thickening of the SPN was found in 13.34% of cases, suggesting that this is a common finding. These findings also highlight the need for adequate clinical information when performing high-resolution ultrasound in the diagnostic workup for nerve pathologies at the dorsum of the foot, especially compression neuropathies.

Focal nerve thickening has been widely studied in symptomatic patients, particularly in the context of trauma or compressive neuropathies. The mechanisms of neuroma formation, especially after amputation, are well established and typically involve axonal disruption, Wallerian degeneration, and subsequent regenerative fibrosis leading to focal enlargement [15].

Todd et al. postulate that the epineurium is thickest in regions where nerves are exposed to mechanical stress, such as near joints or where they cross rigid surfaces, protecting the nerve from damage [16]. This supports the notion that not every nerve thickening should be interpreted as a pathological finding. Physiological variations in nerve size can occur due to local mechanical stress, repetitive motion, or anatomical adaptation.

Other polyneuropathies such as autoimmune neuropathies, including Multifocal Motor Neuropathy and Chronic Inflammatory Demyelinating Polyneuropathy or hereditary neuropathies such as Charcot–Marie–Tooth disease type 1, are known causes of nerve enlargement. They are distinguishable as they are associated with clinical symptoms and typically show multifocal or diffuse nerve enlargement with increased cross-sectional areas at multiple non-entrapment sites on ultrasound [17,18].

Krause et al. divided ATTS into partial ATTS involving either the motor branch to the extensor digitorum brevis or only the sensory branch of the DPN after its division, or as complete ATTS when involving both components [2]. The participants of this study experienced neither of those symptoms. The literature describes several distinct sites of DPN compression within the ATT. In our study, findings such as thickening at the level of the talus and navicular bone appear to correspond with previously reported compression sites in the region of the inferior extensor retinaculum. Similarly, findings near the naviculocuboid joint and cuboid bone align with the crossing of the peroneus brevis tendon, and the TMT joint may correspond to compression by the deep fascia [1,3,19]. Nerve fibrosis and changes in nerve diameter are mostly considered to be caused by mechanical stress [20]. This is supported by the occurrence of nerve thickening at compression sites. Bianchi et al. described ultrasound findings in four surgically confirmed cases of partial ATTS, reporting neuromas of the DPN with a median transverse diameter of 2.7 mm (range: 2.6–3.0 mm) and a median longitudinal length of 4.9 mm (range: 3.8–5.4 mm) [20]. In comparison, the present study found a median CSA of 2.14 mm^2^ (range: 0.84–5.16 mm^2^) and a median length of 3.98 mm (range: 1.46–9.95 mm) for thickened DPN segments in healthy volunteers, suggesting no substantial discrepancy in nerve thickening between asymptomatic individuals and patients with ATTS.

This study is the first to present imaging findings, anatomical localization, and measurements of thickenings of the nerves of the dorsum of the foot in asymptomatic individuals. A study by Symeonidis et al. described asymptomatic nerve thickenings of the interdigital plantar nerves, but without looking at the nerves of the dorsal foot as described in this study [21]. T. Ceri et al. also discusses the topic of nerve diameter changes at potential compression sites without corresponding clinical symptoms but at the upper extremity [22].

To the best of our knowledge, we are the first to evaluate CSA and the length of nerve thickening. We were able to show a correlation between CSA and the length of the increased nerve thickness in DPN, suggesting that these nerve thickenings can be quantified in both ways. However, no clear correlation could be found in SPN, possibly due to the small number of cases. Further studies with a bigger sample size and including symptomatic participants are necessary to reproduce those findings and to determine if there is a CSA threshold to differentiate between asymptomatic and symptomatic neuromas.

We identified fewer thickenings along the distal course of the SPN compared to the DPN. The thickenings that were observed were located at sites that are also anatomically consistent with potential compression points. Thickenings at the level of the talocrural joint were found near the inferior extensor retinaculum, while those at the level of the fibula showed a clear spatial relationship to the bone itself. These locations correspond to published compression zones where ultrasound demonstrates focal nerve enlargement/neuroma; the distribution of deep peroneal nerve thickenings on the dorsum of the foot, including the first tarsometatarsal region and beneath the inferior extensor retinaculum, likewise mirrors prior reports.

While the SN is likely to be injured, possibly due to its superficial course, we could not find any thickenings of the sural nerve at the level of the ankle or the lateral foot. We postulate this might be due to the course of the nerve in the subcutaneous layer with more soft tissue between it and the adjacent bones, and it passing no structures that are usually associated with nerve compression [4,8]. Conversely the sural nerve was not affected, which may be due to its more protected position against mechanical injuries such as those caused by foot and shoe wear.

Considering the number and size of asymptomatic nerve thickenings found in this study, it may be hypothesized that ultrasound imaging of the nerves of the dorsal foot might lead to false-positive results. Thus, a stronger emphasis should be put on clinical examination. Differential diagnoses such as L5 radiculopathy or bone and muscle injury as well as polyneuropathy should be kept in mind for pain syndromes and weakness at the dorsum of the foot. Focal nerve thickening, interpreted as a neuroma in continuity, should only be considered as a potential explanation for clinical symptoms if these cannot be accounted for otherwise. In cases where such a lesion is suspected, targeted local anesthetic nerve blocks may be considered to verify the clinical relevance of the finding [23].

Our findings demonstrate a high prevalence of asymptomatic DPN thickening across all age groups. One limitation of this study is the high count of young participants; however, the consistent prevalence of asymptomatic nerve thickening across all age groups, and especially the high occurrence in a young population, suggests that its occurrence is not age dependent.

Our study is limited by its sample size and age distribution, as it included a predominance of younger individuals. Therefore, percentages derived from subgroup analyses should be interpreted with caution due to limited reliability. Additionally, the cross-sectional design may introduce bias and limit the ability to draw causal inferences. The lack of symptomatic patients for comparison further constrains the interpretability and generalizability of our findings.

## 5. Conclusions

This study found a considerably high prevalence of asymptomatic thickening of the DPN and SPN at the dorsal foot in healthy volunteers of a broad range of ages, highlighting the importance of correlating imaging results with clinical findings. Further studies are needed to compare those findings in an asymptomatic population to individuals with clinical symptoms of ATTS or compression of the SPN to evaluate possible differentiating ultrasound features.

## Figures and Tables

**Table 1:** Participant characteristics.

	Study Participants (*n* = 60)
Age—median (range)	28 (20–78)
female—*n* (%)	33 (55.0)
flatfoot—*n* (%)	1 (1.7)
arthritis—*n* (%)	2 (3.3)
DPN: one-sided thickening—*n* (%)	21(35.0)
DPN: bilateral thickeninigs—*n* (%)	9 (15.0)
SPN: one-sided thickening—*n* (%)	7 (11.7)
SPN: bilateral thickeninigs—*n* (%)	1 (1.7)

**Table 2:** Measurements of thickenings of the deep peroneal nerve, the superficial peroneal nerve and the sural nerve. Abbreviations: CSA = cross-sectional area, DPN = deep peroneal nerve, SPN = superficial peroneal nerve.

	left Foot (*n* = 59)	Right Foot (*n* = 59)	Total Participants (*n* = 60)
Thickenings DPN	*n* = 20 (33.90%)	*n* = 19 (32.20%)	*n* = 30 (50.00%)
CSA (mm^2^)—median (range)	1.93 (0.84–3.66)	2.43 (1.10–5.16)	2.14 (0.84–5.16)
length (mm)—median (range)	3.53 (1.46–8.62)	4.91 (1.62–9.95)	3.98 (1.46–9.95)
Thickenings SPN	4/59 feet—6.78%	5/59 feet—8.47%	8/60 participants—13.33%
CSA medial branch (mm^2^)—median (range)	2.38 (2.1–2.66)	1.22 (1.22–1.22)	1.99 (1.22–2.66)
length medial branch (mm)—median (range)	7.60 (7.21–7.98)	1.85 (1.85–1.85)	5.68 (1.85–7.98)
CSA intermediate branch (mm^2^)—median (range)	1.60 (1.38–1.82)	1.89 (0.68–5.24)	1.82 (0.68–5.24)
length intermediate branch (mm)—median (range)	2.58 (2.24.2.92)	3.05 (2.15–4.69)	3.02 (2.24–4.69)

## Data Availability

The data that support the findings of this study are available from the corresponding author upon reasonable request. Data are not publicly available due to privacy and ethical restrictions.

## Abbreviations

The following abbreviations are used in this manuscript:

ATT	anterior tarsal tunnel
ATTS	anterior tarsal tunnel syndrome
CSA	cross-sectional area
DPN	deep peroneal nerve
SPN	superficial peroneal nerve
SN	sural nerve
TMT	tarsometatarsal
TC	talocrural
IP	Interphalangeal
